# Survey of Treponemal Infections in Free-Ranging and Captive Macaques, 1999–2012

**DOI:** 10.3201/eid2305.161838

**Published:** 2017-05

**Authors:** Amy R. Klegarth, Chigozie A. Ezeonwu, Aida Rompis, Benjamin P.Y.-H. Lee, Nantiya Aggimarangsee, Mukesh Chalise, John Cortes, M. Feeroz, Barbara J. Molini, Bess C. Godornes, Michael Marks, Michael Schillaci, Gregory Engel, Sascha Knauf, Sheila A. Lukehart, Lisa Jones-Engel

**Affiliations:** University of Washington, Seattle, Washington, USA (A.R. Klegarth, C.A. Ezeonwu, B.J. Molini, B.C. Godornes, S.A. Lukehart, L. Jones-Engel);; Udayana University, Denpasar, Bali, Indonesia (A. Rompis);; University of Kent, Canterbury, UK (B.P.Y-H. Lee); Chiang Mai University, Chiang Mai, Thailand (N. Aggimarangsee);; Tribhuvan University, Kirtipu, Nepal (M. Chalise);; HM Government of Gibraltar, Gibraltar, Gibraltar (J. Cortes);; Jahangirnagar University, Dhaka, Bangladesh (M. Feeroz);; London School of Hygiene and Tropical Medicine, London, UK (M. Marks);; University of Toronto Scarborough, Toronto, Ontario, Canada (M. Schillaci);; Samuel Simmonds Memorial Hospital, Barrow, Alaska, USA (G. Engel);; German Primate Center, Gottingen, Germany (S. Knauf)

**Keywords:** Treponema pallidum, Treponema pallidum subsp. pertenue, Macaca spp., yaws, macaques, nonhuman primates, Asia, Southeast Asia, pets, Sulawesi, Indonesia, nontreponemal, bacteria, eradication, mammalian host reservoirs, One Health, surveillance

## Abstract

Survey results showed treponemal infection among pet macaques in Southeast Asia, a region with a high prevalence of human yaws. This finding, along with studies showing treponemal infection in nonhuman primates in Africa, should encourage a One Health approach to yaws eradication and surveillance activities, possibly including monitoring of nonhuman primates in yaws-endemic regions.

Yaws, an endemic tropical disease distinguished by bone and skin lesions, is caused by infection with *Treponema pallidum* subsp. *pertenue* treponemes. Successful yaws treatment campaigns during 1950–1965 were followed by a resurgence of disease, and the World Health Organization (WHO) consequently mounted a yaws eradication campaign (*1*). Although the agent of yaws is spread among humans via direct contact, research has shown that nonhuman primates (NHPs) may serve as mammalian host reservoirs with the potential for zoonotic transmission (*2*). Successful eradication campaigns depend on there being no reservoir shielding the agent from eradication efforts; thus, the role that NHPs play in yaws among humans must be determined (*3*).

African Old World primates (OWPs) can be infected by *T. pallidum* and exhibit symptoms of yaws (*2*). Of note, the *Treponema* Fribourg-Blanc strain (isolated from a baboon in western Africa in 1966) exhibits remarkable genetic similarity to strains that cause yaws in humans (*4*) and in experiments, was shown capable of infecting humans (*5*). More recently, studies focusing on treponemal infections among NHPs in eastern Africa and the Republic of Congo showed that the NHP geographic range overlaps considerably with areas having a formerly high prevalence of yaws in humans (*2*).

Macaques (*Macaca* spp.), OWPs native to Asia and northern Africa, are susceptible to and have been experimentally infected with *T. pallidum* (*6*). After the initial WHO eradication efforts, yaws was believed to be largely eliminated from countries of mainland Asia, although reporting and active case detection have not been uniform throughout the region (*7*). Several island nations in Asia, however, continue to report active human yaws cases (*8*,*9*).

Macaques, the most widely distributed and numerous NHPs in the world, are sympatric with humans throughout Asia, thriving in human-altered environments and commonly kept as pets. To further characterize the role NHPs might play in the maintenance of *T. pallidum* subspecies, we screened an extensive archive of serum samples collected from free-ranging and captive macaques.

## The Study

As part of a project characterizing the pathogen landscape among macaques and humans, we collected blood samples from NHPs during 1999–2012 and stored them at −80°C (*10*). We retrospectively screened samples from 734 macaques representing 13 species distributed throughout the animal’s natural geographic range (Table 1). Study protocols were approved by the University of Washington Institutional Animal Care and Use Committee (no. 4233–01) and adhered to the American Society of Primatologists Principles for the Ethical Treatment of NHPs (https://www.asp.org/society/resolutions/EthicalTreatmentOfNonHumanPrimates.cfm). 

**Table 1:** Number and species of free-ranging and captive macaques tested for treponemal infection, by location, 2000–2014*

Country, species	Year(s) sampled	Total no. sampled	No. captive	No. free-ranging
Nepal	2003			
* Macaca mulatta*		28	0	28
Bangladesh	2008–2012			
* M. mulatta*		137	14	123
Thailand	2003			
* M. arctoides*		2	2	0
* M. assamensis*		5	5	0
* M. fascicularis*		2	2	0
* M. mulatta*		9	9	0
* M. nemestrina*		4	4	0
Cambodia	2011			
* M. fascicularis*		39	0	39
* M. leonina*		5	0	5
* M. nemestrina*		1	0	1
* M. spp. (hybrid)*		3	0	3
Singapore	2003, 2005–2006, 2009			
* M. fascicularis*		76	0	76
Gibraltar	2004, 2009, 2013–2014			
* M. sylvanus*		124	0	124
Indonesia				
Bali	2000–2003			
* M. fascicularis*		157	0	157
Java	2002			
* M. fascicularis*		25	25	0
Sulawesi	2000–2002			
* M. balantak*		5	5	0
* M. fascicularis*		5	5	0
* M. hecki*		7	7	0
* M. maura*		9	9	0
* M. nemestrina*		2	2	0
* M. nigra*		22	14	8
* M. nigrescens*		11	11	0
* M. ochreata*		1	1	0
* M. tonkeana*		40	40	0
*Macaca spp*. (hybrid)		15	15	0
Total	2003–2014	734	170	564

*The 734 tested macaques represented 13 species. Captive category included pets, macaques used in performances, and macaques in zoos; free-ranging included wild macaques, urban macaques, and those at temples, shrines, and reserve parks.

We used a Macro-Vue RPR Card Test Kit (BD, Franklin Lakes, NJ, USA) to screen the 734 blood samples; 11 (1.5%) were positive (Table 2). The RPR (rapid plasma reagin) test, a lipoidal test (nontreponemal) for IgG and IgM typically associated with treponemal infection, can occasionally elicit nonspecific responses. To confirm RPR-positive samples, we used ESPLINE TP (Fujirebio, Tokyo, Japan), an enzyme immunoassay for measuring reactivity to 2 recombinant *T. pallidum* antigens, Tp47 and Tp17. ESPLINE TP and RPR tests have been validated for use in OWPs (*11*). Of the 11 RPR test–positive samples, 1 was from Singapore; 2 from Bali, Indonesia; and 8 from Sulawesi, Indonesia. Six samples (all from Sulawesi) yielded confirmatory positive results on the ESPLINE TP assay. Of note, in Sulawesi, the only positive macaques were pets sampled from South Sulawesi and West Sulawesi Provinces, which make up the island’s southwestern peninsula (Figure). We also used ESPLINE TP to test the 28 RPR test–negative samples from Sulawesi’s southwestern peninsula; none tested positive.

**Table 2:** Treponemal infections in blood samples from free-ranging and pet macaques, by geographic location, 1999–2012*

Location	No. macaques positive/no. negative (% reactive)†	No. macaques tested/no. positive‡
Indonesia		
Bali	2/155 (1.3)	2/0
Java	0/25 (0)	NA
Sulawesi	8/109 (7.3)	8/6
Nepal	0/28 (0)	NA
Singapore	1/75 (1.3)	1/0
Bangladesh	0/137 (0)	NA
Thailand	0/22 (0)	NA
Cambodia	0/48 (0)	NA
Gibraltar	0/124 (0)	NA
Total	11/734 (1.5)	11/0

*NA, indicates that samples in the region were not tested. †Determined by using the Macro-Vue RPR (rapid plasma reagin) test (BD, Franklin Lakes, NJ, USA). ‡Determined by using ESPLINE TP (Fujirebio Inc., Tokyo, Japan), a reagent for the detection of *Treponema pallidum* antibodies.

**Figure:**
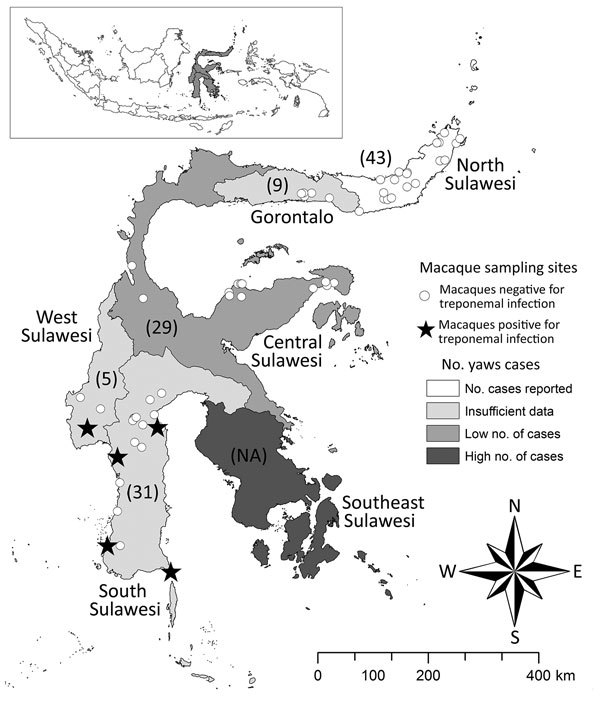
Individual sampling sites where macaques were tested for infection with *Treponema* spp. during 1999–2012 and the number of human yaws cases during 2001–2011, Sulawesi, Indonesia. Numbers in parentheses indicate number nonhuman primates sampled in each of the 6 provinces. ESPLINE TP (Fujirebio Inc., Tokyo, Japan) reagent for the detection of *T. pallidum* antibodies was used to determine whether macaque samples were positive for treponemal infection. The number of human yaws cases was determined by the World Health Organization (*1*). Inset map shows the location of Sulawesi in Indonesia (gray shading). NA, not available.

At the time of sampling, the macaques underwent a physical examination, including close inspection of head, trunk, extremities, oral cavity, and genitals. We conducted a retrospective review of the data and found that none of the macaques had lesions typical of treponemal infection (*4*). Of the 734 macaques, 13, including 2 seropositive macaques from Sulawesi’s southwestern peninsula, had hypopigmentation on the palms of their hands, feet, or both. Hypopigmentation is rarely seen in yaws but is a common manifestation of pinta, which is caused by infection with *T. carateum*, a close relative of *T. p.* *pertenue*.

## Conclusions

Our findings show that pet macaques in Southeast Asia can be infected with *Treponema* spp. related to those that infect humans. The overall prevalence of infection was low in our survey, but the pocket of infection detected among pets in Sulawesi’s southwestern peninsula is noteworthy. The demonstration of reactivity in the serologic tests provides unequivocal evidence that the macaques had been infected with *T. pallidum* or a highly related pathogen. We had hoped to amplify a portion of *tp0548*, a locus in the *T. pallidum* genome used for molecular typing, but no amplifiable pathogen DNA was found in the whole-blood samples that had been held in storage for >10 years. Therefore, we could not determine whether the treponemal strains from NHPs in Sulawesi resembled strains that cause human yaws.

Sulawesi, the third largest island in the Indonesian archipelago, has a population of ≈17 million persons and 7 endemic macaque species. The seropositive samples from South Sulawesi and West Sulawesi Provinces were collected in July and August of 2000, immediately predating an active yaws outbreak among humans in the region that caused 241 documented cases in the neighboring southeastern peninsula during 2001–2011 (WHO, http://apps.who.int/iris/bitstream/10665/75528/1/WHO_HTM_NTD_IDM_2012.2_eng.pdf) (Figure). During that outbreak, WHO characterized the South Sulawesi and West Sulawesi Provinces as “data deficient” regions in regard to the status of yaws among the human population. Most macaques whose samples were used in this study were free-ranging, but all of the macaques sampled in South Sulawesi and West Sulawesi Provinces had been captured at a young age for use as pets. The association between humans and pet macaques is often intimate, with the sharing of food; space; and physical contact through grooming, play, or aggression (*12*). Two of the *Treponema* spp.–infected pets were owned by the same person and housed together. Studies of pet macaques in Sulawesi and their owners have indicated that infectious agents can move between these populations (*12*,*13*). Although the treponemal serologic status of the pet owners in this study is unavailable, the fact that seropositive pet NHPs from a region neighboring an area with a high number of human yaws cases suggests that the NHP cases resulted from treponeme transmission from humans to pets.

All macaques in this study, with the exception of *M. sylvanus* from Gibraltar, were from historically yaws-endemic areas where WHO conducted past yaws eradication campaigns. Much of Asia has a rich tradition of human–NHP commensalism, and macaques are common in villages, often as pets (*10*). Moreover, we previously showed that macaques can harbor an array of mammalian picornaviruses, astroviruses, and mycobacteria (*13*–*15*), underscoring the role of macaques in the ecology of these pathogens. However, as with our current study of treponemal infections, definitive evidence for transmission and the direction of transmission have not been established for these pathogens.

Our findings of treponemal infections among macaques in Southeast Asia, along with published work showing infection in NHPs in Africa (*4*), should encourage holistic and One-Health approaches to eradication and surveillance activities, including consideration of monitoring NHPs in yaws-endemic regions. Such approaches are particularly relevant for pet NHPs, which can easily be assessed and treated. The human–NHP interface is ancient and complex, and continued research, particularly in yaws-endemic regions, can help to ameliorate concerns as a second WHO yaws eradication campaign moves forward.

## References

[1] The World Health Organization. Eradication of yaws—the Morges strategy. Wkly Epidemiol Rec. 2012;87:189–94. 24340400

[2] Knauf S, Liu H, Harper KN. Treponemal infection in nonhuman primates as possible reservoir for human yaws. Emerg Infect Dis. 2013;19:2058–60. 10.3201/eid1912.130863 24274094 PMC3840862

[3] Marks M, Mitjà O, Vestergaard LS, Pillay A, Knauf S, Chen CY, et al. Challenges and key research questions for yaws eradication. Lancet Infect Dis. 2015;15:1220–5. 10.1016/S1473-3099(15)00136-X 26362174 PMC4668588

[4] Zobaníková M, Strouhal M, Mikalová L, Cejková D, Ambrožová L, Pospíšilová P, et al. Whole genome sequence of the *Treponema* Fribourg-Blanc: unspecified simian isolate is highly similar to the yaws subspecies. PLoS Negl Trop Dis. 2013;7:e2172. 10.1371/journal.pntd.0002172 23638193 PMC3630124

[5] Smith JL, David NJ, Indgin S, Israel CW, Levine BM, Justice J Jr, et al. Neuro-ophthalmological study of late yaws and pinta. II. The Caracas project. Br J Vener Dis. 1971;47:226–51. 4936861 10.1136/sti.47.4.226 PMC1048203

[6] Nichols HJ. Experimental yaws in the monkey and rabbit. J Exp Med. 1910;12:616–22. 10.1084/jem.12.5.616 19867348 PMC2124819

[7] Lahariya C, Pradhan SK. Can Southeast Asia eradicate yaws by 2010? Some lessons from the Yaws Eradication Programme of India. Natl Med J India. 2007;20:81–6. 17802987

[8] Mitjà O, Marks M, Konan DJ, Ayelo G, Gonzalez-Beiras C, Boua B, et al. Global epidemiology of yaws: a systematic review. Lancet Glob Health. 2015;3:e324–31. 10.1016/S2214-109X(15)00011-X 26001576 PMC4696519

[9] Hotez PJ, Bottazzi ME, Strych U, Chang LY, Lim YAL, Goodenow MM, et al. Neglected tropical diseases among the Association of Southeast Asian Nations (ASEAN): overview and update. PLoS Negl Trop Dis. 2015;9:e0003575. 10.1371/journal.pntd.0003575 25880767 PMC4400050

[10] Jones-Engel L, Steinkraus KA, Murray SM, Engel GA, Grant R, Aggimarangsee N, et al. Sensitive assays for simian foamy viruses reveal a high prevalence of infection in commensal, free-ranging Asian monkeys. J Virol. 2007;81:7330–7. 10.1128/JVI.00343-07 17475645 PMC1933339

[11] Knauf S, Dahlmann F, Batamuzi EK, Frischmann S, Liu H. Validation of serological tests for the detection of antibodies against *Treponema pallidum* in nonhuman primates. PLoS Negl Trop Dis. 2015;9:e0003637. 10.1371/journal.pntd.0003637 25803295 PMC4372418

[12] Jones-Engel L, Engel GA, Schillaci MA, Babo R, Froehlich J. Detection of antibodies to selected human pathogens among wild and pet macaques (*Macaca tonkeana*) in Sulawesi, Indonesia. Am J Primatol. 2001;54:171–8. 10.1002/ajp.1021 11443632

[13] Wilbur AK, Engel GA, Rompis A, A Putra IG, Lee BP, Aggimarangsee N, et al. From the mouths of monkeys: detection of *Mycobacterium tuberculosis* complex DNA from buccal swabs of synanthropic macaques. Am J Primatol. 2012;74:676–86. 10.1002/ajp.22022 22644580 PMC3368330

[14] Oberste MS, Feeroz MM, Maher K, Nix WA, Engel GA, Hasan KM, et al. Characterizing the picornavirus landscape among synanthropic nonhuman primates in Bangladesh, 2007 to 2008. J Virol. 2013;87:558–71. 10.1128/JVI.00837-12 23097448 PMC3536389

[15] Karlsson EA, Small CT, Freiden P, Feeroz MM, Matsen FA IV, San S, et al. Non-human primates harbor diverse mammalian and avian astroviruses including those associated with human infections. PLoS Pathog. 2015;11:e1005225. 10.1371/journal.ppat.1005225 26571270 PMC4646697

